# Supplementary material for: Effects of Different Exercise Therapies on Balance Function and Functional Walking Ability in Multiple Sclerosis Disease Patients—A Network Meta-Analysis of Randomized Controlled Trials
Source: Int J Environ Res Public Health. 2022 Jun 11;19(12):7175. doi: 10.3390/ijerph19127175 (PMC9222772; doi:10.3390/ijerph19127175)
Supplement: Supplementary file 1 [file ijerph-19-07175-s001.zip › ijerph-1742087-supplementary.pdf]

Supplementary Table S1. Risk of bias for each included studies

Study	Random sequence generation	Allocation concealment	Blinding of participants and personnel	Blinding of outcome assessment	Incomplete outcome data	Selective reporting	Other bias	Overall Score (Category)
Ahmadi	Low	Low	Low	Unclear	Unclear	Low	Low	2(Low)
Ahmadi	Low	Low	High	Unclear	Low	Low	Unclear	3(Moderate)
Gervasoni	High	Unclear	Unclear	Low	Low	Low	Low	3(Moderate)
Straudi	Low	Unclear	Unclear	Unclear	Low	Low	Low	3(Moderate)
Tollar	Low	Low	Low	Unclear	Unclear	Low	Unclear	3(Moderate)
Cakt	Low	Low	Low	Unclear	High	Low	Unclear	3(Moderate)
Orban	Low	Low	Unclear	Unclear	Low	Low	Low	2(Low)
Straudi	Low	Low	Unclear	Unclear	Low	Low	Low	2(Low)
Asvar	Low	Low	Low	Unclear	High	Low	Unclear	3(Moderate)
Gheitasi	Low	Low	Low	High	Low	Low	Low	1(Low)
Gunduz	Low	Low	Unclear	Unclear	Low	Unclear	Low	3(Moderate)
Karlon	Low	Low	Unclear	Unclear	High	Unclear	Low	4(Moderate)
Kara	Low	Unclear	High	High	Unclear	Unclear	Unclear	6(High)
Kucuk	Low	Low	Low	Unclear	Low	Low	Unclear	2(Low)
Zuhal	Low	Low	Low	Unclear	Unclear	Low	Low	1(Low)
Gerson	Low	Unclear	Low	Low	Low	Low	Low	1(Low)
Yazgan	Low	Unclear	Low	Low	High	Low	Low	2(Low)
Khalil	Low	Low	High	Low	Low	Unclear	Low	2(Low)
Brichetto	Low	Low	Unclear	Unclear	High	Unclear	Unclear	5(High)
Lozana	Low	Low	Low	Low	Low	Low	Unclear	1(Low)
Molhemi	Low	Unclear	High	Low	Low	Unclear	Low	3(Moderate)

Tollar	Low	Low	High	Unclear	High	Low	Low	2(Low)
Aidar	Low	Low	Low	High	Low	Low	Unclear	2(Low)
Kargarfard	High	Unclear	Unclear	Unclear	High	Low	Low	5(High)
Aidar	Low	Unclear	Unclear	High	High	Low	Low	5(High)
Moradi	Low	Low	Low	Unclear	Low	Low	Unclear	2(Low)
Moghadasi	Low	Low	High	Unclear	Low	Low	Unclear	3(Moderate)
Alguacil	Low	Low	Low	High	Low	Low	Low	1(Low)
Broekmans	Low	Low	Unclear	Unclear	Low	Unclear	Low	3(Moderate)
Schuhfried	Low	Low	Low	Unclear	High	Unclear	Low	3(Moderate)
Young	Low	Low	High	Unclear	Low	Unclear	Unclear	4(Moderate)

Supplementary Table S2. Consistency test for BBS

	Coef.	Std. Err.	z	P>|z|	[95% Conf. Interval]	
B VS CON	-.5753756	1.984116	-0.29	0.772	-4.464171	3.31342
C VS CON	-4.008412	1.119461	-3.58	0.070	-6.202516	0.27271
D VS CON	-1.303709	1.496103	-0.87	0.384	-4.236018	1.6286
E VS CON	-.5683561	3.30238	-0.17	0.863	-7.040903	5.90419
F VS CON	.1130494	1.517479	0.07	0.941	-2.861155	3.087253
G VS CON	-1.558356	3.088381	-0.50	0.614	-7.611472	4.494759
H VS CON	1.494099	1.745425	0.86	0.392	-1.92687	4.915068

Supplementary Table S3. Consistency test for TUG

	Coef.	Std. Err.	z	P>|z|	[95% Conf. Interval]	
B VS CON	-1.051339	1.73007	-0.61	0.543	-4.442215	2.339536
C VS CON	1.528631	.3975662	3.84	0.120	-0.7494159	2.307847
D VS CON	.1722765	.4755295	0.36	0.717	-0.7597441	1.104297
E VS CON	.7411849	.4727213	1.57	0.117	-.1853318	1.667702
F VS CON	.0791612	.6520774	0.12	0.903	-1.198887	1.357209
G VS CON	.4291348	.625476	0.69	0.493	-.7967755	1.655045
H VS CON	1.228631	4.367539	0.28	0.778	-7.331588	9.78885
